# 培美曲塞治疗全身化疗和吉非替尼治疗失败的肺腺癌32例临床观察

**DOI:** 10.3779/j.issn.1009-3419.2010.08.11

**Published:** 2010-08-20

**Authors:** 标 吴, 诚 黄, 侃 蒋, 武 庄, 振武 徐, 晶 张

**Affiliations:** 350014 福州，福建医科大学教学医院，福建省肿瘤医院内科 Department of Medical Oncology, Fujian Provincial Tumor Hospital, Fujian Medical University Educational Hospital, Fuzhou 350014, China

**Keywords:** 培美曲塞, 肺肿瘤, 化疗, 吉非替尼, Pemetrexed, Lung neoplasms, Chemotherapy, Geftinib

## Abstract

**背景与目的:**

吉非替尼二线或三线治疗晚期非小细胞肺癌的维持时间尚不理想。本研究旨在观察培美曲塞对全身化疗和吉非替尼治疗失败后肺腺癌患者的疗效和毒副反应。

**方法:**

32例经过全身化疗和吉非替尼治疗后肿瘤进展的晚期肺腺癌患者，培美曲塞500 mg/m^2^静脉滴注，第1天，每21天为1周期，并口服地塞米松、叶酸和肌肉注射维生素B_12_以减轻毒副反应。根据实体瘤疗效评价标准对客观缓解率进行评价，毒副反应按美国国立癌症研究所制定的通用药物毒性评价标准（第3版）进行评价。

**结果:**

32例患者中，部分缓解4例（12.5%），疾病稳定11例（34.4%），疾病进展17例（53.1%），中位无进展生存期为2.7个月，中位生存期为11.0个月，1年生存率为37.5%，最常见的毒副反应为骨髓抑制，多为Ⅰ、Ⅱ级。非血液学毒性反应较轻，病人耐受性良好。

**结论:**

对于晚期肺腺癌患者，在化疗和吉非替尼治疗失败后，给予培美曲塞解救治疗有临床获益。

## Abstract (en)

**Background and objective:**

The relapse-free time of gefitinib as the second or third line therapy in patients with advanced non-small cell lung cancer (NSCLC) was not satisfactory. The aim of this study is to evaluate the efficacy and toxicity of pemetrexed in patients with pulmonary adenocarcinoma after failure to chemotherapy and gefitinib.

**Methods:**

A total of 32 relapsed patients with pulmonary adenocarcinoma after failure to chemotherapy and gefitinib received pemetrexed 500 mg/m^2^ by the intravenous administration on the first day, with 21 days as a cycle. Dexamethasone, folic acid and vitamin B_12_ were applied to relieve the drug toxicity. The objective response rate was estimated by Response Evaluation Criteria in Solid Tumors (RECIST) and the toxicity was estimated by National Cancer Institute Common Toxicity Criteria (version 3.0).

**Results:**

For a total of 32 patients, 4 patients reached partial response (PR), constituting a total of 12.5%; 11 patients reached stable disease (SD), constituting a total of 34.4%; 17 patients reached progressive disease (PD), constituting a total of 53.1%. The median progression free survival (PFS) was 2.7 months. The median overall survival (OS) was 11.0 months. One-year survival rate was 37.5%. The most common adverse events (AEs) were myelosuppression with grade Ⅰ and grade Ⅱ toxicity. Other adverse events were tolerated.

**Conclusion:**

Pemetrexed was clinically beneficial for the patients with pulmonary adenocarcinoma after the failure of chemotherapy and gefitinib.

非小细胞肺癌（non-small cell lung cancer, NSCLC）是全球发病率和死亡率较高的恶性肿瘤，其中70%-80%的患者确诊时已属晚期，失去了手术机会，以铂类为基础联合第三代化疗药物组成的一线化疗方案有效率约16%-32%，1年生存率约31%-46%。吉非替尼是表皮生长因子受体酪氨酸激酶抑制剂（epidermal growth factor receptor tyrosine kinase inhibitors, EGFR-TKI），通常作为晚期NSCLC的二线或三线治疗，其疗效与多西紫杉醇二线治疗相近，患者的耐受性更好。但是，吉非替尼治疗晚期NSCLC的维持时间尚不理想^[1]^。接受吉非替尼治疗后疾病进展的患者，目前尚无标准的治疗方案。福建省肿瘤医院内科采用培美曲塞二钠治疗全身化疗和吉非替尼治疗失败的晚期肺腺癌，将其疗效和毒副反应报告如下。

## 1 材料与方法

### 1.1 病例选择

所有患者均经组织学或细胞学证实为肺腺癌；根据2002年美国癌症研究联合会（American Joint Committee on Cancer, AJCC）癌症分期手册（第6版）NSCLC分期标准为Ⅲb期或Ⅳ期；既往经过全身化疗和吉非替尼治疗后肿瘤进展；根据实体瘤疗效评价标准（Response Evaluation Criteria in Solid Tumors, RECIST）^[2]^标准至少有1个可测量的病灶；ECOG评分≤2分；治疗前检查血象、肝肾功能基本正常，无严重心脏病和其它合并症；既往未采用过培美曲塞化疗。

### 1.2 治疗方法

在培美曲塞治疗前1周-2周开始每日口服补充叶酸400 μg，直至最后一次给药后21天。第1次培美曲塞治疗前7天内肌肉注射维生素B_12_ 1 000 μg，以后每9周重复肌注1次。使用培美曲塞前1天、当天和第2天口服地塞米松片4 mg，每日2次。培美曲塞500 mg/m^2^加入生理盐水100 mL中静脉滴注10 min以上，第1天，每21天为1周期，持续应用到肿瘤进展或发生不可耐受的毒副反应。每个治疗周期进行影像学检查以评价疗效。所有患者治疗期间定期检查血常规、肝肾功能和心电图。

### 1.3 疗效评价和毒性评估

疗效评价采用RECIST，分为完全缓解（complete response, CR）、部分缓解（partial response, PR）、疾病稳定（stable disease, SD）和疾病进展（progressive disease, PD），CR和PR在首次评价4周后再进行疗效确认。客观缓解率（objective response rate, ORR）是指CR+PR患者占全组患者的百分率。疾病控制率（disease control rate, DCR）是指CR+PR+SD患者占全组患者的百分率。无进展生存期（progression free survival, PFS）是指患者从首次用药至疾病进展或任何原因死亡的时间。总生存期（overall survival, OS）是指患者首次用药至任何原因死亡的时间。毒性反应按美国国立癌症研究所制定的通用药物毒性反应标准（NCI-CTC 3.0）进行评价。

### 1.4 统计学方法

应用SPSS 16.0统计软件进行统计处理，疗效和临床特征的相关性应用*Fisher*确切概率法检验。PFS和OS采用*Kaplan-Meier*法。双侧检验，以*P* < 0.05为差异具有统计学意义。

## 2 结果

### 2.1 临床特点

2007年6月-2009年12月共有32例患者入组，其中男性23例，女性9例；年龄46岁-71岁，中位年龄63岁；ECOG评分0分-1分29例，2分3例；吸烟者21例，非吸烟者11例；Ⅲb期（伴有胸腔积液）4例，Ⅳ期28例。一线化疗方案中紫杉醇联合铂类23例，长春瑞滨联合铂类5例，吉西他滨联合铂类4例。吉非替尼作为二线治疗13例（40.6%），吉非替尼作为三线治疗19例（59.4%）。既往化疗DCR为46.9%（15/32），既往吉非替尼治疗DCR为43.8%（14/32）。

### 2.2 疗效

32例患者中，无CR病例，PR 4例（12.5%），SD 11例（34.4%），PD 17例（53.1%），ORR为12.5%，DCR为46.9%。患者的临床特征和疗效的相关性见表 1。本组中位PFS为2.7个月（95%CI: 2.187-3.213）（图 1）。随访至2009年12月，32例患者28例已死亡，中位OS为11.0个月（95%CI: 7.742-14.258），1年生存率为37.5%（图 2）。

**1:** 32例肺腺癌患者的临床特征与疗效 Clinical characteristics and efficacy of 32 patients with pulmo-nary adenocarcinoma

Clinical characteristics	*n*	DCR (%)	*P*
Gender			0.243
Male	23	39.1	
Female	9	66.7	
ECOG			> 0.999
0-1	29	48.3	
2	3	33.3	
Smoking status			0.266
No	11	63.6	
Yes	21	38.1	
Stage			0.603
Ⅲb	4	25.0	
Ⅳ	28	46.9	
Response to chemotherapy			0.723
PR+SD	15	53.3	
PD	17	41.2	
Response to gifitinib			0.476
PR+SD	14	57.1	
PD	18	38.9	
DCR: disease control rate; PR: partial response; SD: stable disease; PD: progressive disease.			

**1:**
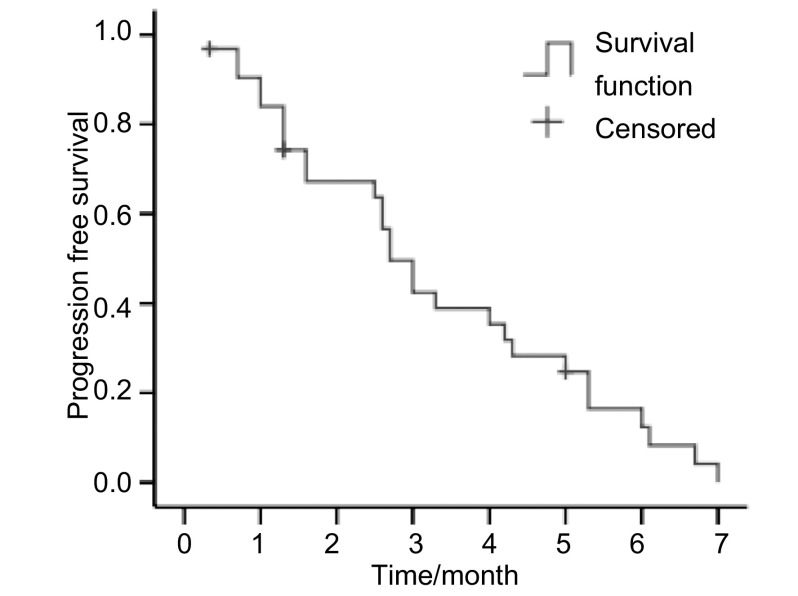
无进展生存时间（*Kaplan-Meier*曲线） Progression free survival (*Kaplan-Meier* curve)

**2:**
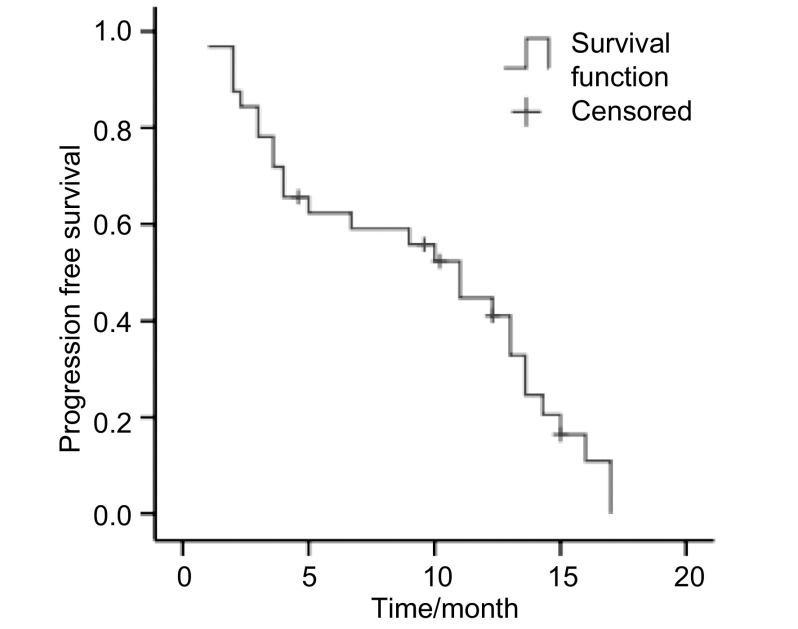
总生存时间（*Kaplan-Meier*曲线） Overall survival (*Kaplan-Meier* curve)

### 2.3 症状改善情况

32例患者中，13例症状改善（40.6%），表现为咳嗽、气促好转，乏力减轻，胸痛、胸闷好转和ECOG体力状态改善，中位症状改善时间为17天。4例PR的患者均有症状改善（100.0%），11例SD患者6例有症状改善（54.6%），17例PD的患者3例有症状改善（17.6%）。*Fisher*确切概率法检验提示：PR+SD患者的症状改善率与PD患者相比，差异有统计学意义（*P*=0.01），提示疾病控制与症状改善有关。

### 2.4 毒副反应

本组最常见的毒副反应为骨髓抑制，以中性粒细胞下降最为常见，血红蛋白和血小板下降较少，多为Ⅰ、Ⅱ级。非血液学毒副反应较轻，主要表现为AST升高、皮疹、乏力、脱发、恶心和尿素氮升高，病人耐受良好，毒副反应具体见表 2。

**2:** 培美曲塞治疗吉非替尼失败后的肺腺癌的毒副反应（例） The toxic side effects of pemetrexed as salvage therapy for patients with pulmonary adenocarcinoma after the failure of gefitinib (*n*)

Toxic side effects	Classification of adverse events			
	Ⅰ	Ⅱ	Ⅲ	Ⅳ
Neutropenia	3	2	2	0
Anemia	4	1	1	0
Thrombocytopenia	2	2	1	0
AST increased	3	1	2	0
Rash	2	1	2	0
Fatigue	3	1	1	0
Alopecia	2	2	0	0
Nausea	2	1	0	0
BUN increased	1	1	0	0

## 3 讨论

吉非替尼是一种喹唑啉类小分子化合物，能够进入肿瘤细胞内，通过阻断细胞信号传导通路中的表皮生长因子受体酪氨酸激酶，从而抑制肿瘤细胞增殖、侵袭、浸润和血管形成，并促进肿瘤细胞凋亡，延长肿瘤患者的生存期^[3, 4]^。吉非替尼通常用于晚期NSCLC患者的二线或三线治疗，其失败后尚无标准的治疗方案。Shukuya等^[5]^报道16例在吉非替尼治疗中达到CR或PR的患者，在疾病进展后给予吉非替尼联合紫杉醇治疗，紫杉醇60 mg/m^2^，第1、8、15天使用，每28天为1周期，吉非替尼250 mg，从紫杉醇第1天开始使用。有效率和和DCR分别为13%和75%，中位PFS和OS分别为4.3个月和8.1个月，毒副反应可以接受。

Wu等^[6]^报道了31例晚期NSCLC患者在化疗和吉非替尼治疗失败后，给予多西紫杉醇进行解救治疗的Ⅱ期临床试验结果，研究发现PR 4例（12.9%），SD 10例（32.3%），PD 17例（54.8%），有效率为12.9%，DCR为45.2%。中位生存期为10个月（95%CI: 5.05-15.08），1年生存率为40.6%。对于二线或三线治疗失败后的患者，进行解救治疗的有效率低，患者的耐受性差，应采用毒副作用较低的药物进行试验性治疗。本研究观察了培美曲塞试验性治疗吉非替尼二线或三线治疗失败后ECOG评分0分-2分的晚期肺腺癌患者的疗效和不良反应。32例患者中，ORR为12.5%，DCR为46.9%，中位PFS为2.7个月，中位生存期为11.0个月，1年生存率为37.5%。

培美曲塞（pemetrexed）是多靶点的叶酸拮抗剂，其主要作用机制是通过抑制胸苷酸合成酶（thymidylate synthetase, TS）、二氢叶酸还原酶（dihydrofolate reductase, DHFR）和甘氨酸核糖核苷甲酰基转移酶（glycinamide ribonucleotide formyltransferase, GARFT），阻断嘧啶和嘌呤的合成，使细胞分裂停止在S期，从而抑制肿瘤细胞的生长^[7]^。Hanna等^[8]^报道的一项对比培美曲塞和多西紫杉醇二线治疗NSCLC Ⅲ期随机临床研究。至少接受过一次化疗的571例Ⅲb/Ⅳ期NSCLC患者随机分为培美曲塞治疗组（283例）和多西紫杉醇治疗组（288例）。结果显示两组的有效率（9.1% *vs* 8.8%）、中位生存期（8.3个月*vs* 7.9个月）和1年生存率（均为29.7%）间均无统计学差异。但是在中性粒细胞下降、粒细胞缺乏性发热和脱发等药物性不良反应方面，培美曲塞治疗组显著低于多西紫杉醇治疗组。本研究的有效率、中位生存期高于报道的培美曲塞作为NSCLC的二线治疗中观察到的结果，可能与我们的病例均为腺癌及ECOG评分较好有关。胸苷酸合成酶TS的表达与培美曲塞治疗NSCLC患者的疗效有关，TS表达低则疗效好，反之则相反，而非鳞癌的表达远低于鳞癌的表达。Ceppi等^[9]^分析了56例晚期NSCLC患者标本中TS的表达与肿瘤病理类型的关系，发现腺癌中TS的表达明显低于其在鳞癌中的表达。体力状态好的NSCLC患者有更好的生存期^[10]^。另外，本研究的病例数少，也可能造成结果会有一定的偏差。

综上所述，对于晚期肺腺癌患者，在化疗和吉非替尼治疗失败后，给予培美曲塞解救治疗有一定的临床获益。
